# SARS-CoV-2 pseudovirus dysregulates hematopoiesis and induces inflammaging of hematopoietic stem and progenitor cells

**DOI:** 10.1038/s12276-025-01416-1

**Published:** 2025-03-03

**Authors:** Dong-Hoon Chae, Hyun Sung Park, Kyoung-Myeon Kim, Aaron Yu, Jae Han Park, Mi-Kyung Oh, Soon Won Choi, Jaechul Ryu, Cynthia E. Dunbar, Hee Min Yoo, Kyung-Rok Yu

**Affiliations:** 1 Department of Agricultural Biotechnology, Research Institute of Agriculture and Life Sciences, Seoul National University, Seoul, Republic of Korea; 2 Translational Stem Cell Biology Branch, National Heart, Lung, and Blood Institute, National Institutes of Health, Bethesda, MD USA; 3 Biometrology Group, Korea Research Institute of Standards and Science, Daejeon, Republic of Korea; 4 Institutes of Convergence Technology, INBCT, Seoul, Republic of Korea; 5 Department of Precision Measurement, University of Science and Technology, Daejeon, Republic of Korea

**Keywords:** Haematopoietic stem cells, Experimental models of disease, Myelopoiesis, Viral infection

## Abstract

Severe acute respiratory syndrome coronavirus 2 (SARS-CoV-2) infection primarily affects the respiratory system but may induce hematological alterations such as anemia, lymphopenia and thrombocytopenia. Previous studies have reported that SARS-CoV-2 efficiently infects hematopoietic stem and progenitor cells (HSPCs); however, the subsequent effects on hematopoiesis and immune reconstitution have not yet been described. Here we evaluated the pathological effects of infection of umbilical-cord-blood-derived HSPCs with the SARS-CoV-2 Omicron variant pseudovirus (PsV). Transcriptomic analysis of Omicron PsV-infected HSPCs revealed the upregulation of genes involved in inflammation, aging and the NLRP3 inflammasome, suggesting a potential trigger of inflammaging. Omicron PsV-infected HSPCs presented decreased numbers of multipotential progenitors (granulocyte‒erythrocyte‒macrophage‒megakaryocyte colony-forming units) ex vivo and repopulated primitive hematopoietic stem cells (Ki-67^−^hCD34^+^ cells) in an HSPC transplantation NOD-scid IL2rγ^null^ mouse model (Omicron mouse). Furthermore, Omicron PsV infection induced myeloid-biased differentiation of HSPCs. Treatment with nanographene oxide, an antiviral agent, partially mitigated the myeloid bias and inflammaging phenotype both in vitro and in vivo. These findings provide insights into the abnormal hematopoietic and immune effects of SARS-CoV-2 infection and highlight potential therapeutic interventions.

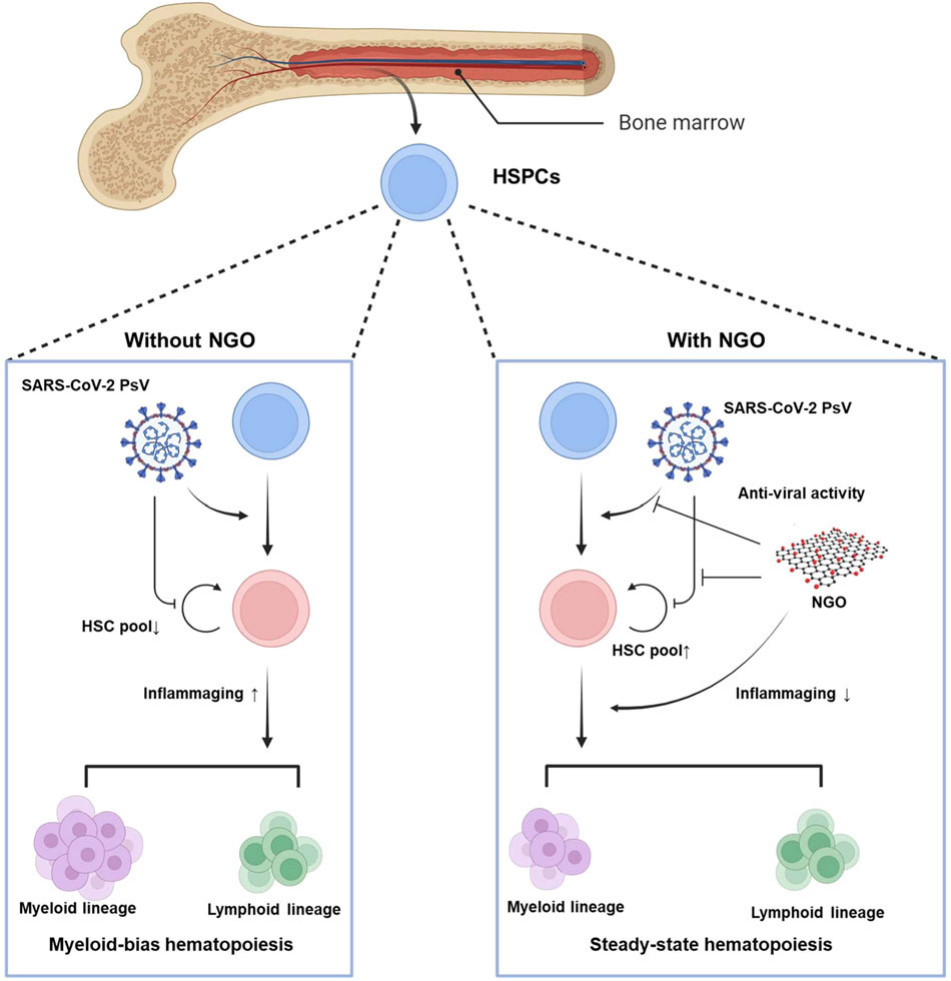

## SARS-CoV-2 alters hematopoiesis and immune response dynamics

The coronavirus disease 2019 (COVID-19) pandemic highlights systemic effects of SARS-CoV-2 beyond respiratory symptoms. Researchers investigated its impact on hematopoietic stem and progenitor cells (HSPCs), which produce blood cells, using a pseudovirus mimicking the Omicron variant. Infection caused inflammation and skewed blood production toward myeloid cells (e.g., macrophages), reducing lymphoid cells (e.g., T and B cells). This imbalance mirrors immune disruptions in COVID-19, such as lymphopenia and excessive inflammation. To address these effects, researchers tested nanographene oxide (NGO), a material with antiviral and anti-inflammatory properties. NGO reduced inflammation and partially restored normal blood production without harming HSPCs. Treated cells showed improved immune balance and reduced myeloid bias. These findings reveal how SARS-CoV-2 disrupts blood cell production and suggest NGO’s potential as a therapeutic tool to manage immune imbalances in COVID-19 and other viral infections. Further studies could explore its broader clinical applications.

This summary was initially drafted using artificial intelligence, then revised and fact-checked by the author.

## Introduction

The rapid propagation and mutation rate of severe acute respiratory syndrome coronavirus 2 (SARS-CoV-2), which is responsible for the worldwide coronavirus disease 2019 (COVID-19) pandemic, has resulted in great loss of human life and social vitality. As of December 2023, 774 million cases and 7 million deaths had been reported to the World Health Organization (https://covid19.who.int/). SARS-CoV-2 has undergone myriad mutations since late 2020, and its contagiousness and risk have changed as a result. SARS-CoV-2 virus-induced infection is initiated by the interaction of viral surface spike proteins with angiotensin-converting enzyme 2 (ACE2), a transmembrane protein expressed on various cell surfaces^1–3^. ACE2 has been reported to be expressed by cerebral neurons, intestinal epithelial cells, renal tubules, immune cells, endothelial cells, red blood cell (RBC) precursors and hematopoietic stem and progenitor cells (HSPCs)^4^. Infection with SARS-CoV-2 through respiratory epithelial cells and other target tissues involves rapid replication of the virus, triggering a severe immune response^3,5,6^. Some patients experience the induction of high levels of proinflammatory cytokines, including TNF family members and IFN-γ, in addition to IL-8, IL-1β, NLRP3 and IL-6. This so-called cytokine storm can damage major tissues such as the lungs, liver, heart and bone marrow (BM) and is associated with a high mortality rate^7–9^. SARS-CoV-2 infection can also affect the hematopoietic system. Histopathological analysis of the BM of COVID-19 decedents revealed hypercellularity and increased immature myelopoiesis. Furthermore, a pattern of severe loss of B cells in the BM or spleen was observed^10^. Additional hematopoietic abnormalities, such as lymphopenia, decreased T cell counts and neutrophilia, have been reported^11–13^. These findings suggest that SARS-CoV-2 infection affects HSPCs either directly, via paracrine or circulating factors, or via an impact on the marrow niche. However, SARS-CoV-2 infection-induced hematopoietic alterations have not been investigated in detail.

Research using live SARS-CoV-2 involves strict biosafety requirements and is restricted to biosafety level 3 laboratories. Pseudovirus (PsV) systems, which utilize particles altered to remove the gene sequences required for viral replication, are safer alternatives for some types of research on SARS-CoV-2 and require less stringent biosafety conditions^14^. In previous studies, SARS-CoV-2 PsVs have been utilized as tools to explore respiratory system infections as well as for the evaluation of vaccines and therapeutic agents^15–18^. We have developed a SARS-CoV-2 PsV system that expresses the spike protein on the viral surface and carries spike protein genomic sequences, enabling this PsV to activate infection signaling pathways through ACE2 and stimulate intracellular spike protein-encoding gene expression^14,19^.

To gain a better understanding of the impact of SARS-CoV-2 on the hematopoietic system, we infected human HSPCs with Omicron PsV and analyzed changes in transcriptomic profiles and HSPC function via lineage reconstitution in vitro and in vivo. We found that the hyperinflammation, cytokine release and lymphopenia observed in COVID-19 may be due in part to an HPSC stress response and dysregulated hematopoiesis. Furthermore, we evaluated the ability of nanographene oxide (NGO) to prevent HSPC alterations caused by Omicron PsV. NGO, a nanoscale graphene oxide, has low cytotoxicity due to its hydrophilic properties derived from the presence of oxygen groups^20,21^. NGO has shown potential in various biomedical settings, including antiviral applications, due to its large surface area, ease of surface functionalization and colloidal stability^22,23^. The antiviral mechanism of NGO is related to oxidative stress induced by functional groups (hydroxyl, epoxy and carboxyl groups) and negative charges, which together damage or inactivate viruses through processes such as photolysis, inhibition of binding and capture^24–27^.

## Methods

### UCB-CD34^+^ cell isolation

Umbilical cord blood (UCB) was obtained from the Seoul Metropolitan Government Public Cord Blood Bank (ALLCORD) in Korea under the approval of the Seoul National University Institutional Review Board (IRB no. E2212/004-001). Human mononuclear cells were isolated as described previously. In brief, UCB was diluted in 1× phosphate-buffered saline (PBS) at a 1:1 ratio, and human mononuclear cells were isolated from the supernatants by Ficoll gradient centrifugation (Ficoll-Paque PLUS, GE Healthcare, 17144003). UCB-derived CD34^+^ HSPCs were isolated using CD34^+^ Microbead kits (Miltenyi Biotech, 130-046-702) according to the manufacturer’s protocol.

### SARS-CoV-2 PsV system production

The green fluorescent protein (GFP)-tagged SARS-CoV-2 *spike* plasmid, psPAX2 packaging vector and pMD2G envelope vector were obtained from the Korea Research Institute of Standards and Science. A SARS-CoV-2 PsV was constructed using ViaFect transfection reagent (Promega). All plasmids were used at 12 µg/ml at the following ratios: SARS-CoV-2 *spike* plasmid or control vector plasmid (6 µg), psPAX2 packaging vector (4.5 µg), pMD2G envelope vector (1.5 µg) and ViaFect (30 µl).

HEK293T cells were seeded at a density of 3 × 10^6^ cells in 7 ml of growth medium and incubated for 24 h at 37 °C with 5% CO_2_. When the cells reached ~80–90% confluence, the plasmids were mixed with 1 ml of Opti-MEM and added to the growth medium for 24 h. The medium was exchanged with 8 ml of fresh growth medium, and media containing viruses were collected at 48 h and 72 h. Collected media containing PsV or the control vector were centrifuged at 300*g* for 10 min to pellet the cell debris, and the supernatant was concentrated via ultracentrifugation at 10,000*g* for 2 h. Concentrated PsV was aliquoted and stored in a −80 °C freezer.

### PsV infection

To evaluate the effects of PsV, HSPCs were treated with PsV at a multiplicity of infection of 1 and 10 µg/µl polybrene (Millipore Sigma) in a 12-well plate. After 24 h of PsV exposure, the medium was replaced with fresh HSPC culture medium, and the cells were cultured for an additional 7 days. Both control-vector-infected and PsV-infected HSPCs were then collected and analyzed by flow cytometry. The expression of viral *spike* sequences was assessed using quantitative reverse-transcription PCR (qRT‒PCR). The HSPC culture medium consisted of X-Vivo 10 serum-free hematopoietic cell medium (Lonza) supplemented with human stem cell factor (100 ng/ml), human FLT3-ligand (100 ng/ml), human thrombopoietin (100 ng/ml), human interleukin-3 (20 ng/ml), human interleukin-6 (20 ng/ml; all from Peprotech) and primocin (25 µg/ml; InvivoGen).

### Preparation, characterization and application of NGO

NGO samples were obtained from INBCT Co., Ltd. (Seoul, Korea) and resuspended in deionized water. These NGOs were synthesized from graphite utilizing Taylor–Couette flow. The morphology of the synthesized NGO particles was characterized using a Cs-corrected high-resolution transmission electron microscope (JEM-ARM200F, Cold FEG, JEOL), with the NGO loaded onto 400-mesh carbon-coated copper grids. The particle size distribution was assessed using a disc centrifuge (CPS Instruments). Further morphological examination was conducted with atomic force microscopy on NGO samples deposited on sapphire wafers in noncontact mode (scanned area: 25 μm², XE-100, Park Systems).

For the study of viral interactions, HSPC cultures infected with Omicron PsV were treated with or without 5 μg/ml NGO for 24 h. Next, the culture media were replaced with fresh media, and the cells were cultured for an additional 7 days. In vivo assessments were conducted by transplanting Omicron PsV-infected HSPC cultures treated under the same conditions into busulfan-conditioned NOD-scid IL2rγ^null^ (NSG) mice. After a period of 12 weeks, the mice were euthanized, and the BM, peripheral blood (PB) and spleen were collected for subsequent analyses.

### CFU assays

For colony-forming assays, HSPCs were seeded at a density of 10^3^ cells with Methocult (Stemcell) in six-well plates. After 2 weeks of incubation, the dishes were examined to determine the number and shape of erythroid burst-forming units (BFU-E), granulocyte‒macrophage colony-forming units (CFU-GM) and granulocyte‒erythrocyte‒macrophage‒megakaryocyte colony-forming units (CFU-GEMM). Colonies were counted manually under an inverted light microscope (Olympus).

### In vitro HSPC lineage differentiation

To identify HSPC lineage differentiation, GFP- or PsV-infected HSPCs were seeded at a density of 10^5^ cells in HSPC culture medium. The cells were collected on day 7 and washed twice in 1× PBS. The cells were stained with Lin-PerCP-Cy5.5, CD34-BV421, CD38-PE-Cy7, CD45RA-APC, CD90-BV510 and CD135-PE (all provided by BD Bioscience) in flow cytometry buffer (2% FBS + 1× PBS) and analyzed on an Attune NxT system (Thermo Fisher Scientific) via FlowJo software. The flow cytometry gating strategy is described in Fig. 3d (left).

### In vitro differentiation of CD71^+^ RBC progenitors

HSPCs infected with PsV were cultured at a density of 1 × 10^5^ cells/ml in HSPC culture medium. The cells were supplemented with 50 U/ml erythropoietin (provided by Peprotech), and fresh medium was added on day 3 of culture. After 7 days, the cells were collected and washed twice with 1× PBS. For the identification of CD71^+^ cells, the samples were stained with CD71-PerCP-Cy5.5 (supplied by BioLegend) dissolved in flow cytometry buffer (2% fetal bovine serum in 1× PBS). Flow cytometric analysis was performed using the Attune NxT system (Thermo Fisher Scientific), and the data were analyzed using FlowJo software.

### In vivo HSPC engraftment

Four-week-old female NSG mice were purchased from JA Bio Industry. All of the mice were maintained under semispecific pathogen-free conditions. For HSPC transplantation, busulfan (Sigma-Aldrich) was dissolved in dimethyl sulfoxide (16 mg/ml) and diluted with PBS at a 1:4 ratio. Diluted busulfan was injected at 20 mg/kg intraperitoneally into the mice. Twenty-four hours later, the HSPCs were resuspended in saline (1 × 10^5^ cells in 200 µl) and injected intravenously into the mice. The mice were cared for until week 12. All animal experiments were conducted under the approved guidelines of Seoul National University’s Institutional Animal Care and Use Committee (SNU-201120-1-3).

### PB and BM isolation

After 12 weeks, the mice were euthanized, and PB and BM were collected. PB was collected as ophthalmic blood and mixed with 5 ml of 1× RBC lysis buffer (BioLegend) for 5 min to remove RBCs. Then, 10 ml of cold PBS was added to the PB samples, followed by centrifugation at 300*g* for 10 min. PB cells were stained with anti-human (h)CD45 (BV421, BD Bioscience) and anti-mCD45 (PE-Cy7, BD Bioscience) antibodies, and the distributions of human and mouse blood cells were analyzed by flow cytometry.

To prepare mouse BM cells, mouse femurs were washed in Dulbecco’s modified Eagle medium, and the suspension was filtered through a 40-μm cell strainer. BM cells were mixed with 1× RBC lysis buffer for 5 min, and the mixture was centrifuged at 300*g* for 10 min. BM cells were stained with anti-hCD45, anti-mouse (m)CD45, anti-hCD34 (BV421, BD Bioscience) and anti-Ki-67 (PE, Thermo Fisher Scientific) antibodies according to the manufacturer’s protocols, and populations of human hematopoietic stem cells (HSCs) were analyzed by flow cytometry.

### RNA sequencing

RNA sequencing was performed by Theragen Bio using Illumina technology as described previously with modifications^28^. Total RNA was extracted and purified from PsV-infected HSPCs studied in vitro or from the BM of engrafted mice using TRIzol reagent (Invitrogen). Libraries were generated using the Illumina TruSeq strand mRNA sample preparation kit (Illumina) and sequenced using the NovaSeq 6000 platform (2 × 150 paired-end sequencing, Illumina) according to the manufacturer’s protocol. After the adapter sequences were removed and low-quality reads were filtered via an in-house script, the filtered reads were aligned to the *Homo sapiens* (human) genome assembly GRCh38 (hg38) using HISAT2. The aligned reads were counted using featureCounts. For differential expression analysis, the gene expression of each group was quantified in R with the edgeR package. Next, differentially expressed genes (DEGs) in control vector- versus Omicron PsV-infected hHSPCs were identified on the basis of an absolute log_2_-fold change ≥0.58 and a false discovery rate <0.05. Then, heat maps were generated using an in-house script, and clustering analysis was performed using a hierarchical clustering method. Volcano plots of DEGs were generated using ggplot 2 in R.

### Gene Ontology and gene set enrichment analysis

DEGs were subjected to gene enrichment analysis with the R package clusterProfiler, and gene set enrichment analysis (GSEA) was performed using the Broad GSEA application. The significance of the gene sets was calculated using GSEA v3.0 (https://www.gsea-msigdb.org/gsea/index.jsp). The significance of each factor was calculated using Fisher’s exact test.

### Pathway analysis

Trimmed DEGs (log_2_-fold change >2.4, false discovery rate <0.05) were used for pathway analysis using QIAGEN’s ingenuity pathway analysis (IPA, QIAGEN, www.qiagen.com/ingenuity).

### Quantitative real-time PCR

Total RNA was obtained using TRIzol reagent, and 1 µg of RNA was converted to cDNA using M-MLV reverse transcriptase (Promega). Real-time PCR was performed using Topreal qPCR 2× premix SYBR Green with low ROX (6-Carboxyl-X-Rhodamine) as a passive reference dye (Enzynomics) and measured using the CFX96 Real-Time PCR system (Bio-Rad). *GAPDH* was used as the reference gene for normalization. The sequences of the primers used for qRT‒PCR are listed in Tables 1 and 2.

**Table 1:** List of human primers used in qRT‒PCR.

Gene	Forward primer (5′-3′)	Reverse primer (3′-5′)
*GAPDH*	GACAGTCAGCCGCATCTTCT	GCGCCCAATACGACCAAATC
*spike*	CCTCATGGAGTGGTGTTCCT	CCTCTGGGTCACAAACCAGT
*IL1R*	ACTCCTCGCTCGGCTCCTA	AGAGGGTGCGTCTACCTGGA
*IL1β*	CCACAGACCTTCCAGGAGAATG	GTGCAGTTCAGTGATCGTACAGG
*IL4*	CCG TAA CAG ACA TCT TTG CTG CC	GAG TGT CCT TCT CAT GGT GGC T
*IL6*	AGACAGCCACTCACCTCTTCAG	TTCTGCCAGTGCCTCTTTGCTG
*IFNα*	GCCTCGCCCTTTGCTTTACT	GCTTGGGATGAGACCCTCCTA
*IFNβ*	TAGCACTGGCTGGAATGAG	GTTTCGGAGGTAACCTGTAAG
*IFNγ*	AGCAGCACCAGTAAGAGGGAGGACTT	TCAAATATTGCAGGCAGGATGACCA
*TNF*	CAGAGGGAAGAGTTCCCCAG	CCTTGGTCTGGTAGGAGACG
*TGFβ1*	GATGTCACCGGAGTTGTGCG	GCCGGTAGTGAACCCGTTGAT
*NLRP3*	GGACTGAAGCACCTGTTGTGCA	TCCTGAGTCTCCCAAGGCATTC
*PYCARD*	AGCTCACCGCTAACGTGCTGC	CTTGGCTGCCGACTGAGGAG
*AhR*	GTCGTCTAAGGTGTCTGCTGGA	CGCAAACAAAGCCAACTGAGGTG
*AhRR*	CACCAGTCTGTGCGAATCGGAA	CAGTCTGTTCCCTGAGCACCAA
*CYP1A1*	GATTGAGCACTGTCAGGAGAAGC	ATGAGGCTCCAGGAGATAGCAG
*IDO*	GCCTGATCTCATAGAGTCTGGC	TGCATCCCAGAACTAGACGTGC
*NRF2*	CACATCCAGTCAGAAACCAGTGG	GGAATGTCTGCGCCAAAAGCTG
*p16*	CTCGTGCTGATGCTACTGAGGA	GGTCGGCGCAGTTGGGCTCC
*CD86*	TGGAGAGGGAAGAGAGTGAACA	GCCCATAAGTGTGCTCTGAA
*FLT3*	AGACTGTCGCTGGGTCCAAGAT	GGAGATGTTGGTCTGGACGAAG
*IL7Rα*	TGGTCATCTTGGCCTGTGTGTT	TCCACCCTATGAATCTGGCAGT
*Notch1*	GCAGACTATGCCTGCAGCTGTG	CGGCACTTGTACTCCGTCAGC
*RUNX1*	CCACCTACCACAGAGCCATCAA	TTCACTGAGCCGCTCGGAAAAG
*vWF*	GGCTTTATCTCCCCCAGCAGT	AATGAGGGCTGCGGCTATCT
*ITGB3*	TGTACCACGCGTACTGACAC	CACTTCTCACAGGTGTCCCC
*PU.1*	GACACGGATCTATACCAACGCC	CCGTGAAGTTGTTCTCGGCGAA
*GM-CSF*	CTGGAGGTCAAACATTTCTGAGAT	GGAGCATGTGAATGCCATCCAG
*CD150*	CCTCTCCTTGACCTTCGTGC	TTCCCAACTGCCGGAGAATC
*Fil1*	GCTGCGAGGTCAGGCTGTAA	CCGTAGTCAGGACTCCCCGA

**Table 2:** List of mouse primers used in qRT‒PCR.

Gene	Forward primer (5′-3′)	Reverse primer (3′-5′)
*GAPDH*	ATGCCAGTGAGCTTCCCGTTCAG	CATCACTGCCACCCAGAAGACTG
*SCF*	ATCTGCGGGAATCCTGTGACTG	CCATATCTCGTAGCCAACAATGAC
*CXCL12*	GCATCAGTGACGGTAAACCA	GTTTAAAGCTTTCTCCAGGTACTC
*VCAM1*	GCTATGAGGATGGAAGACTCTGG	ACTTGTGCAGCCACCTGAGATC

### Statistical analysis

All experiments were performed at least in triplicate. Where the data were normally distributed, the significance of the differences in values between groups was determined using Student’s *t*-tests or one-way or two-way analysis of variance, followed by Tukey’s multiple comparisons test. The data are presented as the mean ± standard deviation. Statistical analyses were performed with GraphPad Prism software (version 8.0.1), and *P* values less than 0.05 were considered statistically significant.

## Results

### Infection by SARS-CoV-2 PsV triggers an immune response in CD34^+^ HSPCs

**Fig. 1:**
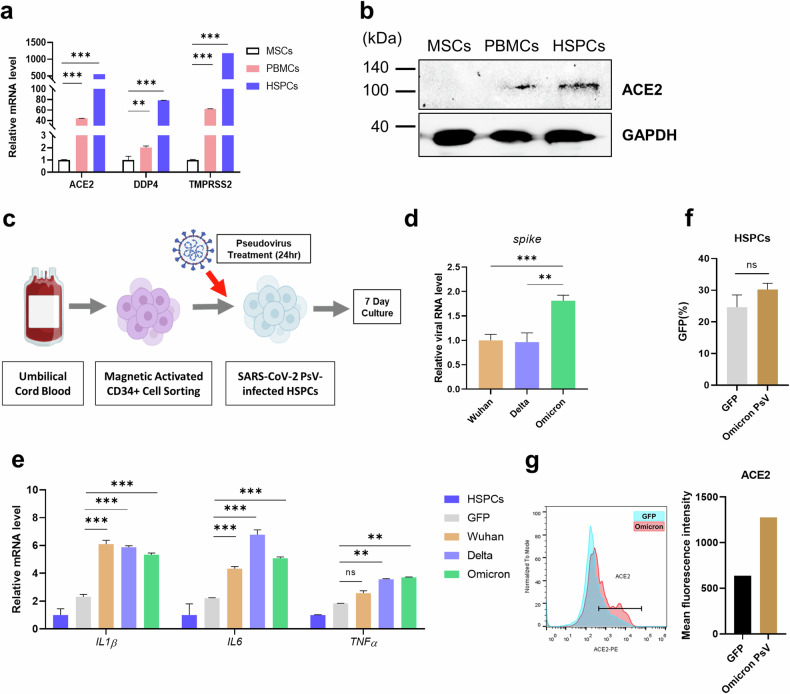
HSPCs are susceptible to SARS-CoV-2 PsV infection. **a**, Expression levels of the SARS-CoV-2 entry-related genes *ACE2*, *DDP4* and *TMPRSS2* in three different cell lines were determined by quantitative RT‒PCR (qRT‒PCR). **b** The protein level of ACE2 in MSCs, PBMCs and HSPCs was determined by western blotting. **c** Scheme of SARS-CoV-2 PsV infection. **d** SARS-CoV-2 PsV infection was confirmed through the detection of the viral *spike* sequence via qRT‒PCR. HSPCs were treated with the SARS-CoV-2 variants PsV, Wuhan, Delta and Omicron. **e** Differences between variants were determined through the relative expression levels of inflammatory genes. Gene expression of *IL1β, IL6* and *TNF* in HSPCs infected with SARS-CoV-2 variants was determined by qRT‒PCR. **f** Flow cytometry analysis of GFP- and Omicron PsV-infected HSPCs. **g** The mean fluorescence intensity of ACE2 expression in GFP- or Omicron PsV-infected HSPCs was quantified using flow cytometry. Relative mRNA levels were normalized to those of *GAPDH*. *P < 0.05, **P < 0.01, ***P < 0.001. ns, not significant. The results are presented as the mean ± s.e.m.

We evaluated the expression of SARS-CoV-2 entry-related genes in mesenchymal stem cells (MSCs), peripheral blood mononuclear cells (PBMCs) and HSPCs to examine their susceptibility to SARS-CoV-2 infection. *ACE2*, *DPP4* and *TMPRSS2* (transmembrane protease serine 2) were more highly expressed in UCB-derived CD34^+^ HSPCs than in PBMCs. MSCs presented minimal expression of *ACE2*, *DDP4* and *TMPRSS2* (Fig. 1a). Similarly, the expression of the SARS-CoV-2 entry receptor ACE2 was confirmed at the protein level in HSPCs (Fig. 1b).

To examine the functional impact of exposure of HSPCs to SARS-CoV-2, we constructed a GFP-tagged SARS-CoV-2 PsV with a SARS-CoV-2 receptor binding domain that expressed the spike protein on the surface of the virus particles. For PsV infection, UCB-derived HSPCs were infected with PsV for 24 h and subsequently cultured for 7 days (Fig. 1c). We generated variants of SARS-CoV-2, such as Wuhan, Delta and Omicron, using the corresponding receptor binding domains to evaluate the functional response of infected cells to each variant. The infection rates of the variants were determined by elevated expression of the viral *spike* RNA (Fig. 1d). All three variants caused an upregulated inflammatory response, as confirmed by the expression of *IL1β, IL6* and *TNF* (Fig. 1e). All further experiments utilized Omicron PsV, reflecting its proximity to currently circulating strains. The multiplicity of infection was adjusted to target approximately 20% GFP positivity for GFP or Omicron PsV (Fig. 1f). Flow cytometry analysis revealed elevated expression levels of ACE2 in HSPCs after infection with Omicron PsV (Fig. 1g). Together, these data suggest that HSPCs express high levels of SARS-CoV-2 entry-related genes and that the engineered PsV expresses the spike protein on its surface and is able to enter and induce a proinflammatory response in HSPCs.

### RNA expression of Omicron PsV-infected HSPCs

**Fig. 2:**
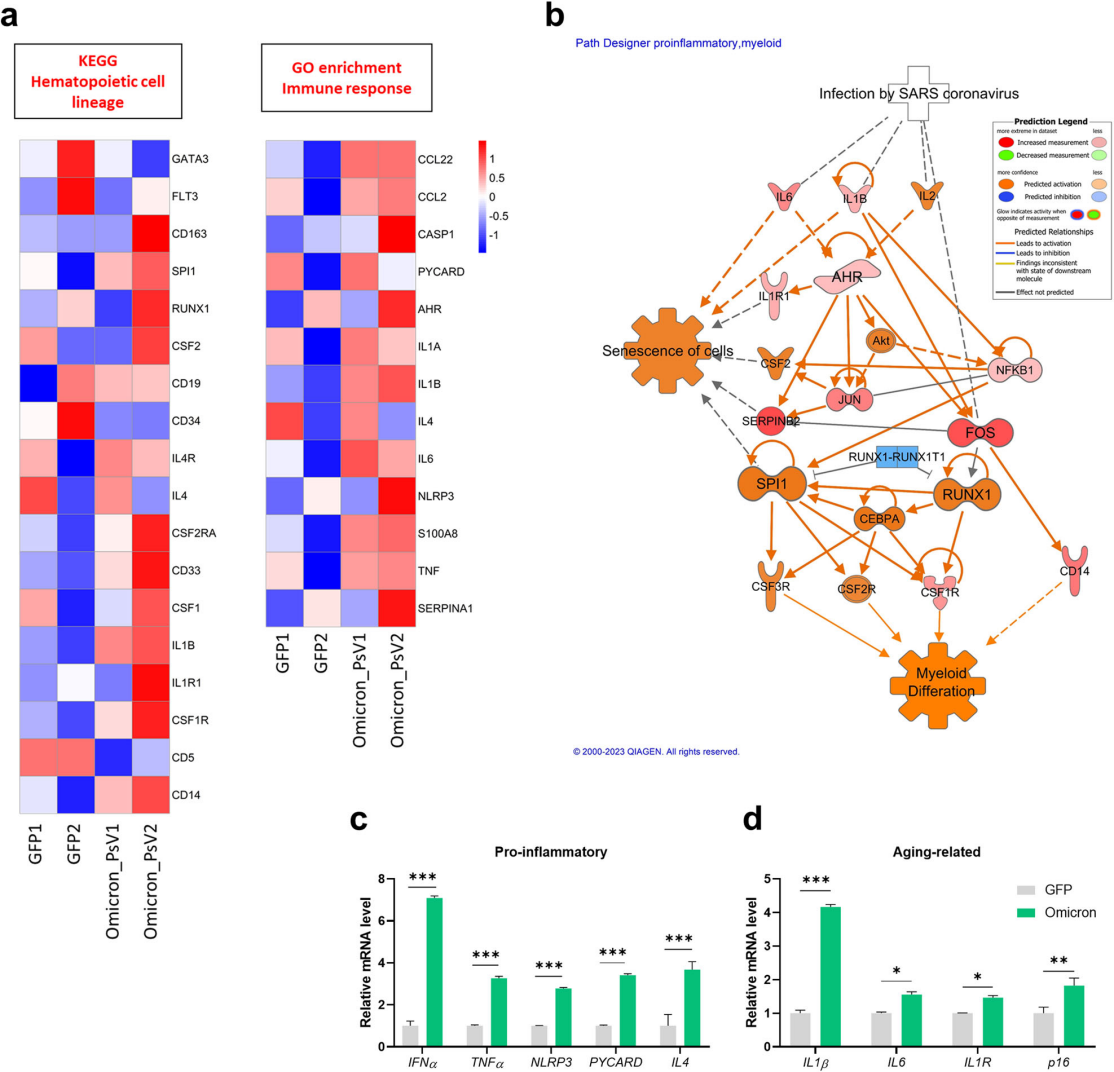
The transcriptomic profiles of Omicron PsV-infected HSPCs reveals a shift toward myeloid differentiation and inflammaging. **a** Heat map of Kyoto Encyclopedia of Genes and Genomes (KEGG) pathway analysis and GO enrichment analysis of DEG profiles in Omicron PsV-infected HSPCs compared with those in GFP-infected HSPCs. **b** IPA of pathways enriched in Omicron PsV-infected HSPCs compared with those enriched in GFP-infected HSPCs. Transcriptional levels of pro-inflammatory (**c**) and aging-related (**d**) genes were determined via qRT‒PCR. Relative mRNA levels were normalized to those of *GAPDH*. *P < 0.05, **P < 0.01, ***P < 0.001. The results are presented as the mean ± s.e.m.

Transcriptomic changes in ex vivo HSPCs after 7 days of Omicron PsV infection were analyzed by bulk RNA sequencing. A total of 1,397 DEGs were identified in Omicron PsV-infected HSPCs compared with GFP-infected control HSPCs (Supplementary Fig. 1a). GSEA of the DEGs revealed changes in genes linked to hematopoietic cell lineage alterations and immune response changes (Fig. 2a). Omicron PsV-infected HSPCs presented upregulated expression of genes related to myeloid differentiation (*CD14*, *CSF1* and *CD33*) and inflammation (*TNF*, *S100A8*, *NLRP3* and *IL-1β*). Subsequent canonical pathway analysis (IPA) revealed the upregulation of pathways related to the senescence of cells, myeloid differentiation and the NLRP3 inflammasome in Omicron PsV-infected HSPCs (Fig. 2b and Supplementary Fig. 1b). We confirmed that the expression of proinflammatory genes (*IFNα*, *TNF*, *NLPR3*, *PYCARD* and *IL4*), aging-related genes (*IL1β*, *IL1R*, *IL6* and *p16*) and reactive oxygen species (ROS)-related genes (*AHR*, *CYP1A1*, *AHRR*, *IDO* and *NRF2*) was increased in Omicron PsV-infected HSPCs compared with GFP control-infected HSPCs via qRT‒PCR (Fig. 2c, d and Supplementary Fig. 1c). These findings indicate that Omicron PsV infection increases the gene expression of proinflammatory-, aging- and ROS-related genes in HSPCs, possibly triggering ‘inflammaging’, inflammatory changes associated with aging.

### Omicron PsV infection induces myeloid-biased hematopoiesis in vitro

**Fig. 3:**
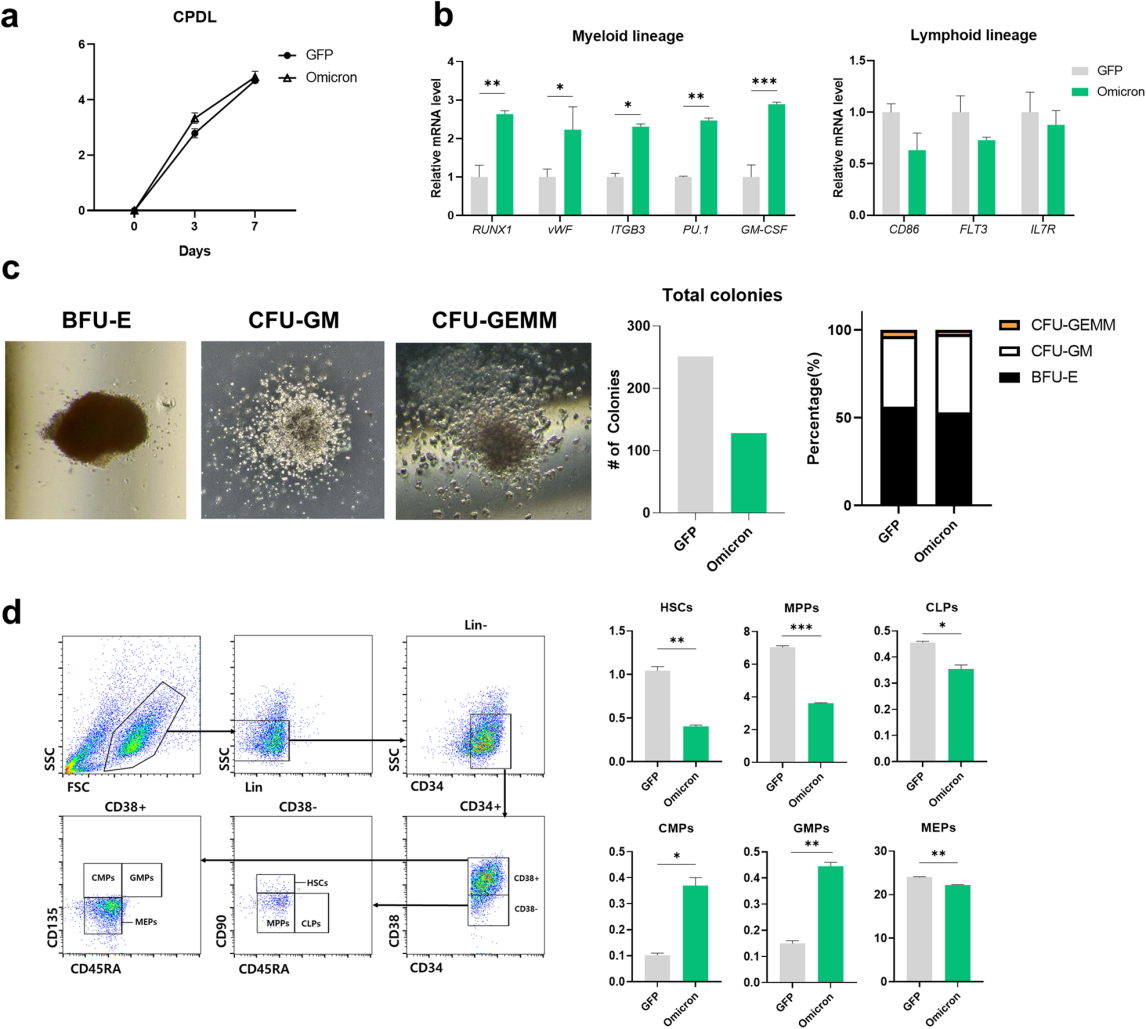
Omicron PsV infection induces a myeloid-biased differentiation phenotype in vitro. **a** Cumulative population doubling levels (CPDLs) of GFP- and Omicron PsV-infected HSPCs at day 3 and day 7. **b** Transcriptional levels of myeloid/lymphoid lineage-related genes in PsV-infected HSPCs were determined by qRT‒PCR. Relative mRNA levels were normalized to those of *GAPDH*. **c** Left: epresentative colony morphologies in the CFU assays. Right: numbers of BFU-E, CFU-GM, and CFU-GEMM and the total sum of all colonies. **d** Right: hematopoietic lineage differentiation was investigated via the expression of lineage-specific markers through flow cytometry. Left: a representative example of flow cytometry gating used to analyze HSPC lineage differentiation. Percentages of HSPC lineage differentiation fractions within Omicron PsV-infected HSPCs. *P < 0.05, **P < 0.01, ***P < 0.001. The results are presented as the mean ± s.e.m.

To identify the physiological effects of Omicron PsV infection using the signaling pathways identified by RNA sequencing, we examined the lineage commitment of Omicron PsV-infected HSPCs. The cumulative population doubling level did not significantly change the expansion of Omicron PsV-infected HSPCs (Fig. 3a). The transcript levels of the myeloid lineage-related genes *RUNX1, vWF*, *ITGB3*, *PU.1* and *GM-CSF* were significantly increased by 7 days of Omicron PsV infection. By contrast, the expression of lymphoid lineage-related genes was not altered upon Omicron PsV infection (Fig. 3b).

Next, we analyzed the number of CFUs formed by GFP-infected versus Omicron PsV-infected HSPCs to evaluate lineage differentiation. Compared with control (GFP-infected) HSPCs, Omicron PsV-infected HSPCs presented notably fewer multipotential progenitors (CFU-GEMM) and erythroid progenitors (BFU-E) and greater numbers of granulocyte‒macrophage progenitors (CFU-GM) (Fig. 3c).

To further confirm changes in cell surface markers related to lineage differentiation, we performed flow cytometric analysis 7 days after GFP or Omicron PsV infection. There were significantly fewer HSCs (Lin^−^CD34^+^CD38^−^CD45RA^−^CD90^+^), multipotent progenitor cells (MPPs, Lin^−^CD34^+^CD38^−^CD45RA^−^CD90^−^), common lymphoid progenitors (CLPs, Lin^−^CD34^+^CD38^−^CD45RA^+^CD90^−^) and megakaryocyte–erythroid progenitors (MEPs, Lin^−^CD34^+^CD38^+^CD45RA^−^CD135^−^) among Omicron PsV-infected cells than among control-infected cells. By contrast, the proportions of common myeloid progenitors (CMPs, Lin^−^CD34^+^CD38^+^CD45RA^−^CD135^+^) and granulocyte‒macrophage progenitors (GMPs, Lin^−^CD34^+^CD38^+^CD45RA^+^CD135^+^) were significantly greater than those of control cells (Fig. 3d). These data indicate that Omicron PsV infection induces myeloid-biased differentiation of HSPCs.

### Omicron PsV induces inflammaging-associated immune remodeling in HSPCs in vivo

**Fig. 4:**
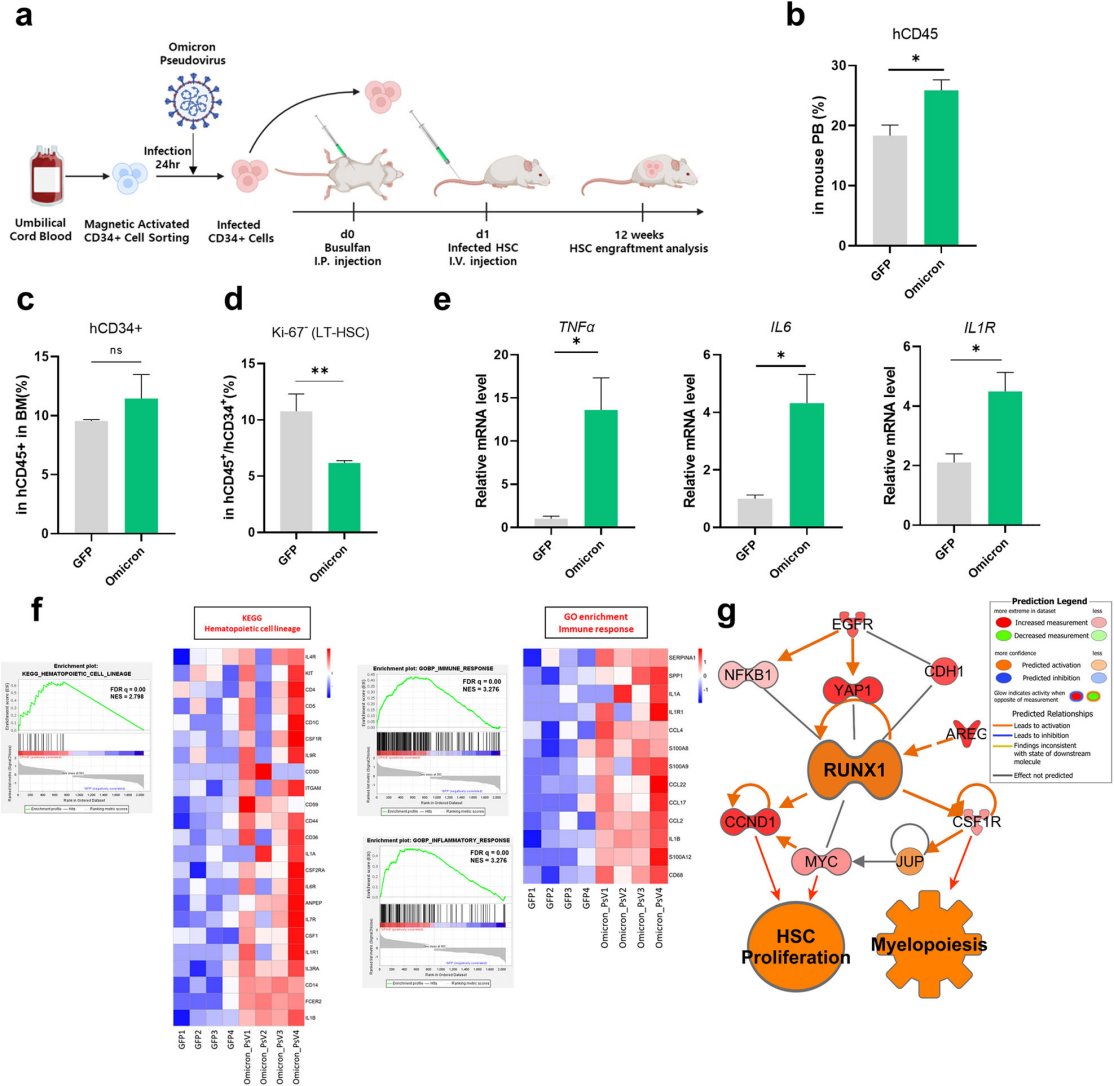
Hematopoietic reconstitution of NSG mice transplanted with Omicron PsV-infected HSPCs reveals inflammaging and myeloid bias. **a**, Schematic representation of the in vivo experiment. This diagram illustrates the transplantation of Omicron PsV-infected HSPCs into NSG mice with and without NGO treatment (*n* = 6 per group). **b** Assessment of engraftment levels (hCD45^+^) via flow cytometry in mouse PB 12 weeks after transplantation. **c**, **d** Percentages of hCD34^+^ cells (**c**) and LT-HSCs (**d**) in mouse BM were determined by flow cytometry. **e** Transcription levels of inflammaging markers in mouse BM were determined by qRT‒PCR. Relative mRNA levels were normalized to those of *GAPDH*. **f** RNA sequencing was performed to evaluate the phenotype of BM human cells in mice transplanted with Omicron PsV-infected HSPCs. KEGG pathway analysis and GO enrichment analysis of DEGs. **g** IPA analysis of BM cells in mice transplanted with Omicron PsV-infected HSPCs. *P < 0.05, **P < 0.01, ***P < 0.001. The results are shown as the mean ± s.e.m.

To evaluate the impact of Omicron PsV on in vivo hematopoiesis, we established an NSG mouse model through the transplantation of Omicron PsV-infected HSPCs (Omicron mice). UCB-derived CD34^+^ cells were cultured with Omicron PsV for 24 h and then transplanted into busulfan-conditioned NSG mice (Fig. 4a). The mice were euthanized after 12 weeks, and the BM, PB and spleen were collected. We confirmed that viral RNA levels of the spike protein were twofold greater in Omicron mouse BM than in control mouse BM by 12 weeks post-transplantation (Supplementary Fig. 2a). The levels of CD45^+^ circulating human blood cells in mouse PB samples were slightly greater in the Omicron mouse group than in the control group (Fig. 4b). The percentage of hCD34^+^ cells was similar in the BM of experimental and control mice (Fig. 4c); however, the number of noncycling Ki-67^−^hCD34^+^ cells (long-term (LT)-HSCs) was decreased in Omicron mice, a phenotype that includes more primitive quiescent HSCs (Fig. 4d). The transcription levels of inflammation- and inflammasome-related genes, including *TNF*, *IL6* and *IL1R*, were elevated in the BM cells of mice transplanted with Omicron PsV-infected HSPCs (Fig. 4e). This pattern of inflammatory gene activation was also observed in the BM and spleen tissues of Omicron mice (Supplementary Fig. 2b,c).

To gain a comprehensive understanding of the transcriptomic changes in the BM of Omicron mice, we conducted RNA sequencing of the marrow 12 weeks after transplantation. GSEA of the DEGs revealed hematopoietic cell lineage alterations, and we further confirmed that the expression of myeloid lineage-related genes (*IL1R1*, *CSF1R*, *CSF1*, *CD36* and *CD14*) was significantly upregulated in Omicron versus GFP mice (Fig. 4f and Supplementary Fig. 2d,e). Furthermore, immune and inflammatory response alterations were identified via Gene Ontology (GO) enrichment analysis, and the expression of inflammation-related genes (*CD68*, *IL1β*, *CCL2*, *S100A8* and *SERPINA1*) was increased in the Omicron group compared with the control group. IPA revealed increased HSPC proliferation, myelopoiesis and inflammation canonical pathways in the Omicron mouse group (Fig. 4g and Supplementary Fig. 2f). These findings indicate that mice transplanted with Omicron-infected PsV-HSPCs exhibit a myeloid bias and an inflammaging phenotype.

### NGO ameliorates Omicron PsV-induced alterations

**Fig. 5:**
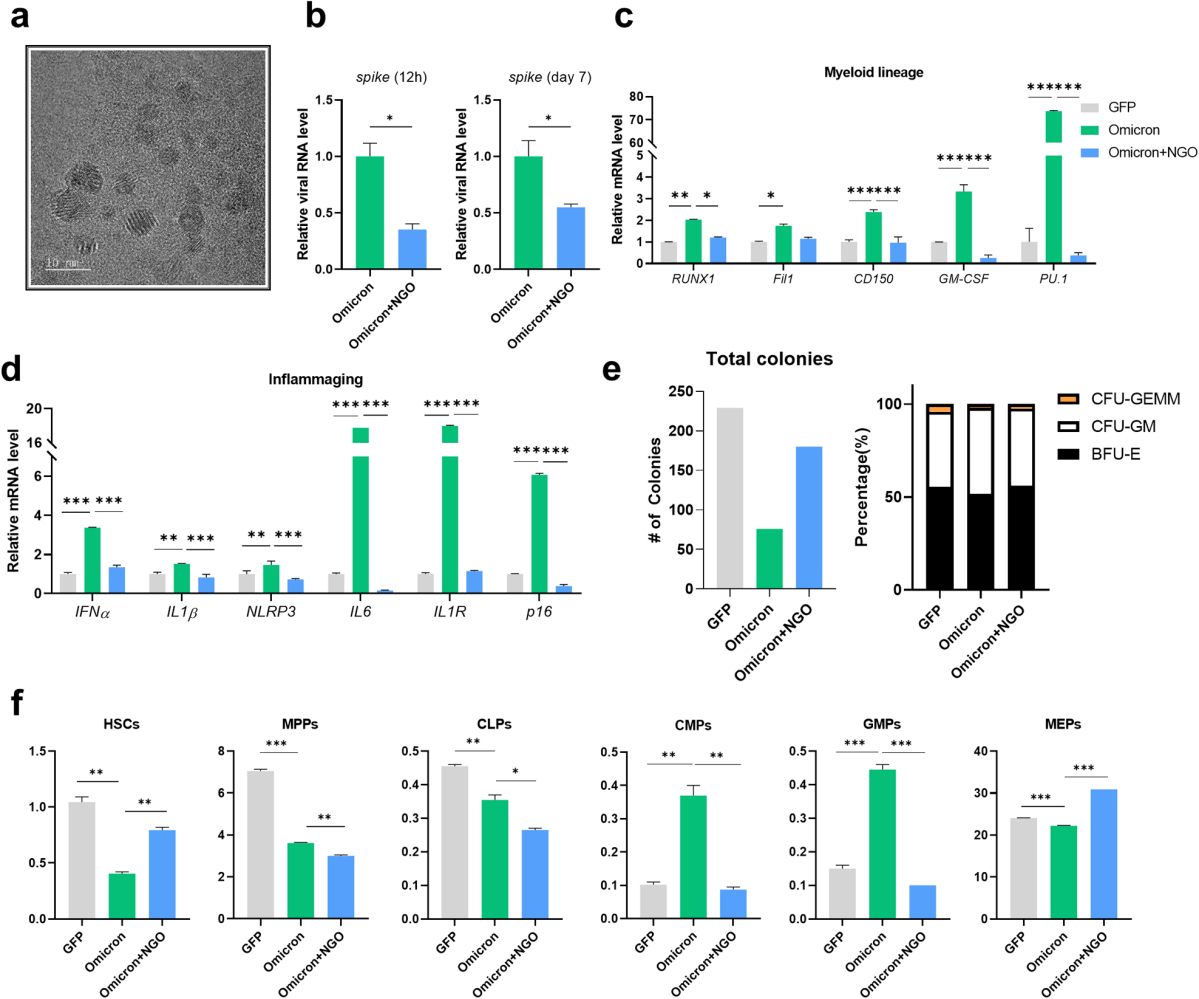
NGO shows antiviral activity against Omicron PsV-infected HSPCs. **a**, Morphologies and sizes of NGO particles were analyzed by TEM. **b** Viral *spike* RNA expression in PsV-infected HSPCs treated with or without NGO was quantified at 12 h and 7 days via qRT‒PCR. **c**, **d**, Transcriptional levels of myeloid lineage-related (**c**) and inflammaging-related (**d**) genes in Omicron PsV-infected HSPCs were determined via qRT‒PCR. Relative mRNA levels were normalized to those of *GAPDH*. **e** A CFU assay was performed, and the numbers of BFU-E, CFU-GM and CFU-GEMM and the total sum of all colonies were quantified. **f** Hematopoietic lineage differentiation was investigated via the expression of lineage-specific markers through flow cytometry. The percentages of HSPC lineage differentiation fractions in the Omicron PsV- and NGO-treated groups are shown. *P < 0.05, **P < 0.01, ***P < 0.001. The results are shown as the mean ± s.e.m.

To examine the effects of NGO on the impact of SARS-CoV-2 spike protein infection on HSPCs, we treated PsV-infected HSPCs with NGO for 24 h, followed by a 7-day culture period. The morphology of the synthesized NGO was confirmed by transmission electron microscopy (TEM) (Fig. 5a), which revealed that the size distribution of the synthesized NGO particles was between 5 nm and 33.7 nm, with an average size of 10 nm (Supplementary Fig. 3a). We first examined the effect of NGO treatment on the proliferative ability of PsV-infected HSPCs. NGO treatment of PsV-infected HSPCs did not affect PsV-infected HSPC expansion, indicating minimal cytotoxicity (Supplementary Fig. 3b). At the RNA level, viral *spike* gene expression was downregulated by NGO treatment at 12 h and 7 days, indicating that NGO treatment partially inhibited *spike* RNA infection by the virus (Fig. 5b). Furthermore, genes associated with inflammaging (*IFNα*, *IL1β*, *NLRP3*, *IL6*, *IL1R* and *p16*) and myeloid lineage (*RUNX1*, *Fil1*, *CD150*, *PU.1* and *GM-CSF*) genes that were upregulated by Omicron PsV infection were downregulated by NGO treatment (Fig. 5c, d). The expression of lymphoid lineage-related genes (*CD86*, *FLT3*, *IL7R* and *Notch1*) remained downregulated during the 7 days of NGO treatment (Supplementary Fig. 3c). NGO treatment of GFP-infected control HSPCs did not result in significant changes in the expression levels of any of the genes we evaluated (Supplementary Fig. 3d). Total colony counts were notably decreased in Omicron-infected HSPCs but recovered in Omicron-NGO-infected HSPCs (Fig. 5e, left). The changes in Omicron-infected HSPCs in terms of BFU-E, CFU-GEMM and CFU-GM were similar to those shown in Fig. 3c, but the proportions of CFU-GEMM and CFU-GM decreased in response to NGO treatment (Fig. 5e, right). Flow cytometric analysis revealed that Omicron-infected HSPCs presented a reverse phenotype in the myeloid lineage (GMP, CMP and MEP) and long-term HSC populations. Unexpectedly, the proportions of the lymphoid lineage (CLP) and MPP populations remained low (Fig. 5f). NGO treatment of GFP-infected HSPCs did not significantly change the cellular morphology or lineage differentiation pattern (Supplementary Fig. 3e). However, we confirmed an increased rate of differentiation of Omicron PsV-infected HSPCs into CD71^+^ RBC progenitors compared with those infected with GFP. Furthermore, this rate of differentiation decreased with NGO treatment (Supplementary Fig. 3f). These results demonstrate that NGO treatment affects hematopoietic lineage differentiation and may reduce hematopoietic perturbations caused by SARS-CoV-2 PsV infection.

**Fig. 6:**
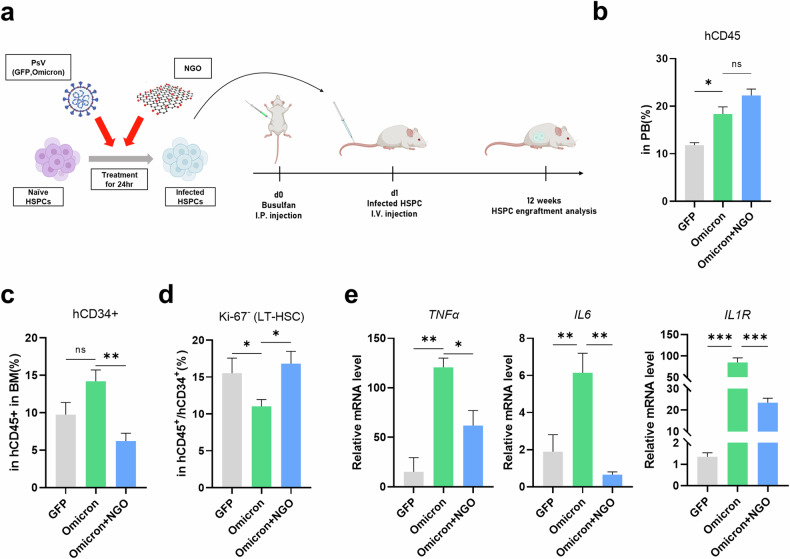
Partial restoration of the inflammaging signature in NSG mice transplanted with Omicron PsV-infected HSPCs treated with NGO. **a** Schematic of the in vivo experiment depicting NSG mice transplanted with Omicron PsV-infected HSPCs, with groups either receiving NGO treatment or left untreated (*n* = 5–7 per group). **b** Engraftment levels (hCD45^+^) in mouse PB were quantified using flow cytometry to compare the treated and untreated groups. **c**, **d** Percentages of hCD34^+^ cells (**c**) and LT-HSCs (**d**) in the BM were assessed by flow cytometry, and both treatment conditions were compared. **e** Transcription levels of inflammaging markers in the BM were quantified via qRT‒PCR to evaluate the effects of NGO treatment versus no treatment. Relative mRNA levels were normalized to those of *hGAPDH*. *P < 0.05, **P < 0.01, ***P < 0.001. The results are shown as the mean ± s.e.m.

In addition, to assess the impact of NGO on Omicron PsV-infected HSPCs in vivo, we utilized an NSG mouse model transplanted with either Omicron PsV-infected HSPCs or Omicron PsV/NGO-treated HSPCs (Fig. 6a). After 12 weeks, the mice were euthanized, and BM and PB were collected. CD45^+^ circulating human blood cells were elevated in both the Omicron PsV and Omicron PsV/NGO mice (Fig. 6b). However, NGO treatment resulted in a decreased percentage of hCD34^+^ cells in the BM (Fig. 6c), but decreased numbers of noncycling Ki-67^−^hCD34^+^ cells (LT-HSCs) in Omicron PsV mice were restored to normal in the NGO-treated group (Fig. 6d). Furthermore, while the expression of inflammation/inflammasome-related genes (*TNF*, *IL6* and *IL1R*) was upregulated in the BM cells of Omicron PsV mice, it was reduced in NGO-treated mice (Fig. 6e). Furthermore, the levels of the BM niche factors *SCF*, *CXCL12* and *VCAM1*, which were decreased in Omicron PsV-treated mice, returned to normal in the NGO-treated group (Supplementary Fig. 3g). These findings suggest that NGO treatment can reverse the abnormal HSPC reconstitution pattern and mitigate the heightened inflammatory response in the BM triggered by Omicron PsV infection.

## Discussion

Under homeostatic conditions or steady-state hematopoiesis, HSPCs reside in the BM in a quiescent state and produce balanced amounts of myeloid and lymphoid progeny. However, the hematopoietic system can be activated for proliferation and differentiation in response to increased demand or stress induced by blood loss or pathological damage such as viral infection^29,30^. Hepatitis C virus, human herpesvirus and human immunodeficiency virus can infect HSPCs and alter hematopoiesis^31–33^. Viral infection can modulate stress-state hematopoiesis by directly targeting HSPCs or through signals released by pathogens such as viral pathogen-associated molecular patterns recognized by pattern recognition receptors, leading to increased expression of proinflammatory cytokines such as *IFNα*, *IL6*, *IL1β* and *GM-CSF*^34–36^. Our SARS-CoV-2 PsV system expresses a GFP-tagged spike protein on the virus surface and was designed to harbor the spike protein in its genome. This system allows us to investigate the interaction between the spike protein and the SARS-CoV-2 entry receptor ACE2, as well as the impact of spike protein-encoding gene expression on hematopoiesis.

SARS-CoV-2 has been reported to cause systemic infections and has been identified in the BM^12,37,38^. Infection of the BM by SARS-CoV-2 increases the expression of *ACE2* mRNA in BM cells, increasing their susceptibility to infection by this virus^39^. Consistent with these previous findings, we confirmed that ACE2 was expressed by HSPCs and that SARS-CoV-2 PsV infection increased the expression of ACE2 in HSPCs (Fig. 1g), suggesting that HSPCs are susceptible to SARS-CoV-2 PsV. We confirmed that viral *spike* RNA levels and the expression of inflammatory genes were elevated in Omicron PsV-infected HSPCs (Fig. 1d, e). Interestingly, IPA of pathways enriched in Omicron PsV-infected HSPCs revealed increased expression of inflammatory factors, elevated intracellular ROS and a decrease in the HSC pool and myelopoiesis, resembling the inflammaging phenotype of HSPCs^30,40,41^.

After the transplantation of Omicron PsV-infected HSPCs into NSG mice, we observed a reduction in the percentage of primitive HSCs (Fig. 4d), altered expression of immune response- and hematopoietic function-related genes (Fig. 4e, f) and increased inflammatory gene expression in the spleen, where immune cells accumulate (Supplementary Fig. 2c). Together with the clinical features of lymphopenia and myelopoiesis/neutrophilia in patients with COVID-19^42,43^, our data suggest that the Omicron spike protein induces the NLRP3 inflammasome and inflammaging signaling and dysregulated myelopoiesis. These molecular signaling changes were observed not only in Omicron PsV-infected HSPCs but also in surrounding uninfected cells, underscoring the complexity of the impact of the virus. This highlights both the direct effects on infected HSPCs and the indirect effects mediated by bystander cells. However, further studies are needed to better understand the effects of the spike protein on the hematopoietic niche and its interaction with HSPCs because of the limitations of generalizing our findings regarding the murine hematopoietic niche in a humanized mouse model to humans.

Furthermore, while the 7-day post-infection in vitro period and 12-week post-transplantation in vivo timeframe may not have directly replicated the acute phase of natural SARS-CoV-2 infection, these intervals were chosen to evaluate the mechanistic impacts of Omicron PsV on HSPCs and hematopoiesis. These periods allow us to observe more sustained changes in hematopoietic and immune functions, potentially due to viral effects or therapeutic interventions. Our observations extend to the impact of SARS-CoV-2 on specific cell lineages. Notably, the Omicron variant, despite its lesser impact relative to earlier strains^44,45^, still increases the number of CD71^+^ erythroid progenitors, indicating a variant-specific influence on erythropoiesis. This response may reflect an erythropoietic adaptation to infection-induced stress, which is relevant to the clinical features of COVID-19, such as anemia. The depletion of B cells, as reported in recent studies^46^, correlates with our findings of myelopoiesis and underscores the broad impact of viruses on immune regulation. This systemic effect on both myeloid and lymphoid lineages emphasizes the importance of a holistic approach to understanding the impact of COVID-19 on hematopoiesis.

The previous use of NGO to treat SARS-CoV-2 infection involved exploiting the hydrophilic properties of NGO to surround the virus before infection or attaching other substances to the NGO particles to act as decoys, minimizing contact with entry receptors on cells^26,47–50^. In this study, we explored the antiviral functions of NGO against Omicron PsV infection. The viral *spike* RNA level was 50% lower in the NGO-treated group than in the control group, suggesting that NGO was effective at preventing Omicron PsV from infecting HSPCs (Fig. 5b). Furthermore, the NGO-treated group presented significantly decreased expression of myeloid lineage genes (*RUNX1*, *PU.1* and *CD150*) and inflammaging-related genes (*IL1β*, *NLRP3*, *IL6*, *IL1R* and *p16*) as well as myeloid-biased differentiation of HPSCs compared with the untreated control group (Fig. 5c, d), possibly due to the anti-inflammatory properties of NGO^51–53^. Interestingly, treatment with NGO appeared to restore the abnormally altered populations of LT-HSCs and CD34^+^ cells, as well as the activated inflammaging observed in the BM of mice transplanted with Omicron PsV-infected HSPCs. These findings, depicted in Fig. 6, suggest that NGO may contribute to the reestablishment of hematopoietic homeostasis. Further research is necessary to comprehensively assess the impact of SARS-CoV-2 infection on dormant HSCs and other hematopoietic cell subsets.

Although the BM microenvironment and cells are not primary targets of SARS-CoV-2, recent studies have demonstrated that human BM cells are prone to SARS-CoV-2 infection and that infection can profoundly alter the hematopoietic system^11,39^. We confirmed that human HSPCs were susceptible to spike protein-induced signaling and demonstrated a significant increase in the expression of inflammaging-related genes followed by altered hematopoiesis, such as the activation of myelopoiesis, in response to Omicron PsV infection. Furthermore, we evaluated the antiviral effects of NGO and showed that it has anti-inflammatory and antiviral properties against Omicron PsV-infected HSPCs. Our data highlight the potential of therapeutics that can address the inflammaging response and imbalanced differentiation of HSPCs to treat SARS-CoV-2 infection.

## Supplementary information

Supplementary Figs. 1–3

## Data Availability

The RNA sequencing data supporting the results of this study have been deposited in the NCBI GEO database under accession number GSE261419.
