# Supplementary material for: SARS-CoV-2 pseudovirus dysregulates hematopoiesis and induces inflammaging of hematopoietic stem and progenitor cells
Source: Exp Mol Med. 2025 Mar 3;57(3):616–27. doi: 10.1038/s12276-025-01416-1 (PMC11958793; doi:10.1038/s12276-025-01416-1)
Supplement: Supplementary file 1 — Supplementary Figs. 1–3 [file 12276_2025_1416_MOESM1_ESM.pdf]

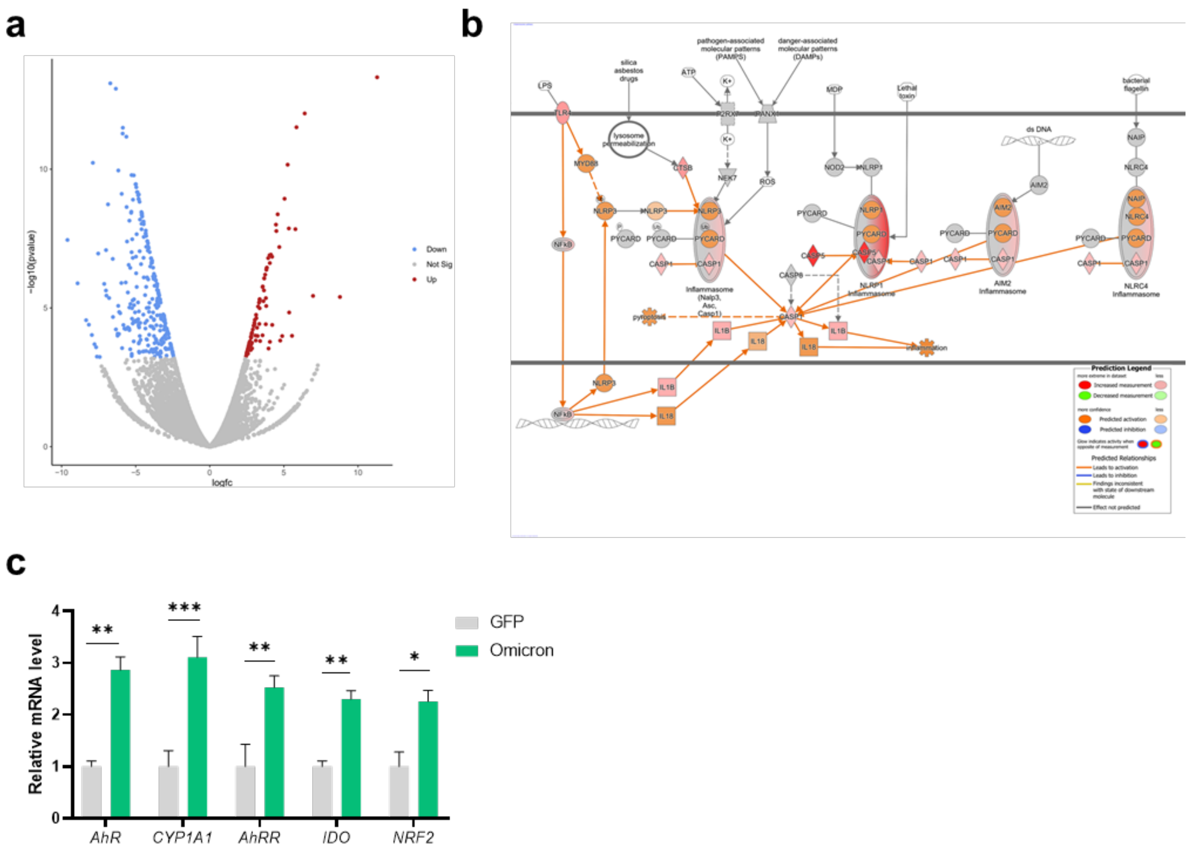

Supplementary Fig. 1. (a) A volcano plot highlighting DEGs in Omicron PsV-infected HSPCs compared to GFP-infected HSPCs. (b) IPA of inflammation/NLRP3-related signals in Omicron PsV-infected HSPCs compared to GFP-infected HSPCs is shown. (c) Transcriptional levels of ROS-related factor in Omicron PsV-infected HSPCs were determined by qRT-PCR. mRNA levels were normalized to *GAPDH*. *, p<0.05; **, p<0.01; ***, p<0.001. Results are represented as the means ± SEM.

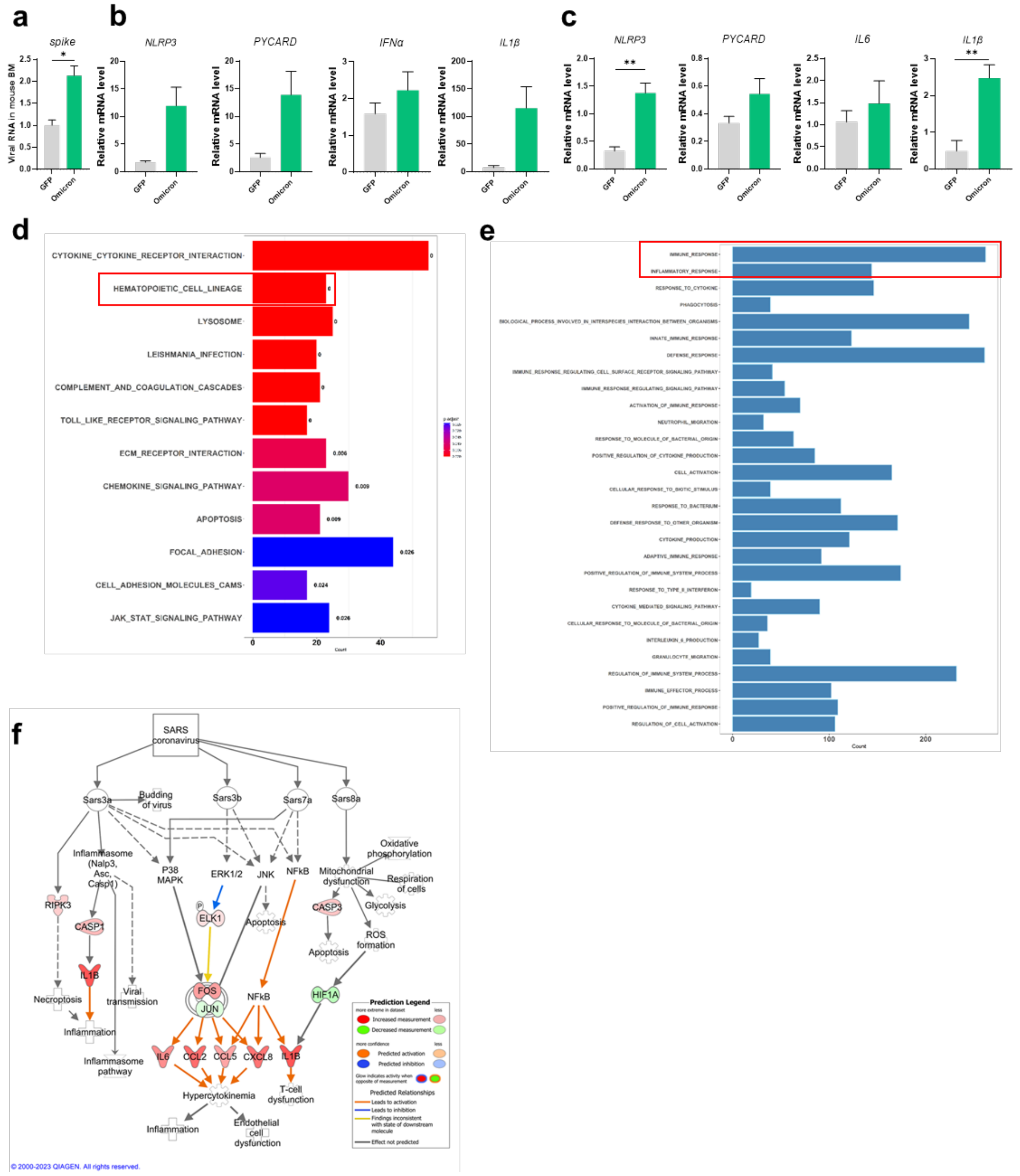

Supplementary Fig. 2. (a-c) Transcription levels of viral RNA (a), pro-inflammatory genes and BM niche related factors (b) in bone marrow and pro-inflammatory genes in spleen (c) were determined by qRT-PCR. mRNA levels were normalized to *GAPDH*. (d, e) RNA sequencing was performed to evaluate the phenotype of BM human cells in NSG mice transplanted with Omicron PsV-infected HSPCs. (d) KEGG pathway analysis and (e) GO

enrichment analysis of the DEGs. (f) IPA analysis of BM human cells in NSG mice transplanted with Omicron PsV-infected HSPCs. *, p<0.05; **, p<0.01; ***, p<0.001. Results are shown as mean ± SEM.

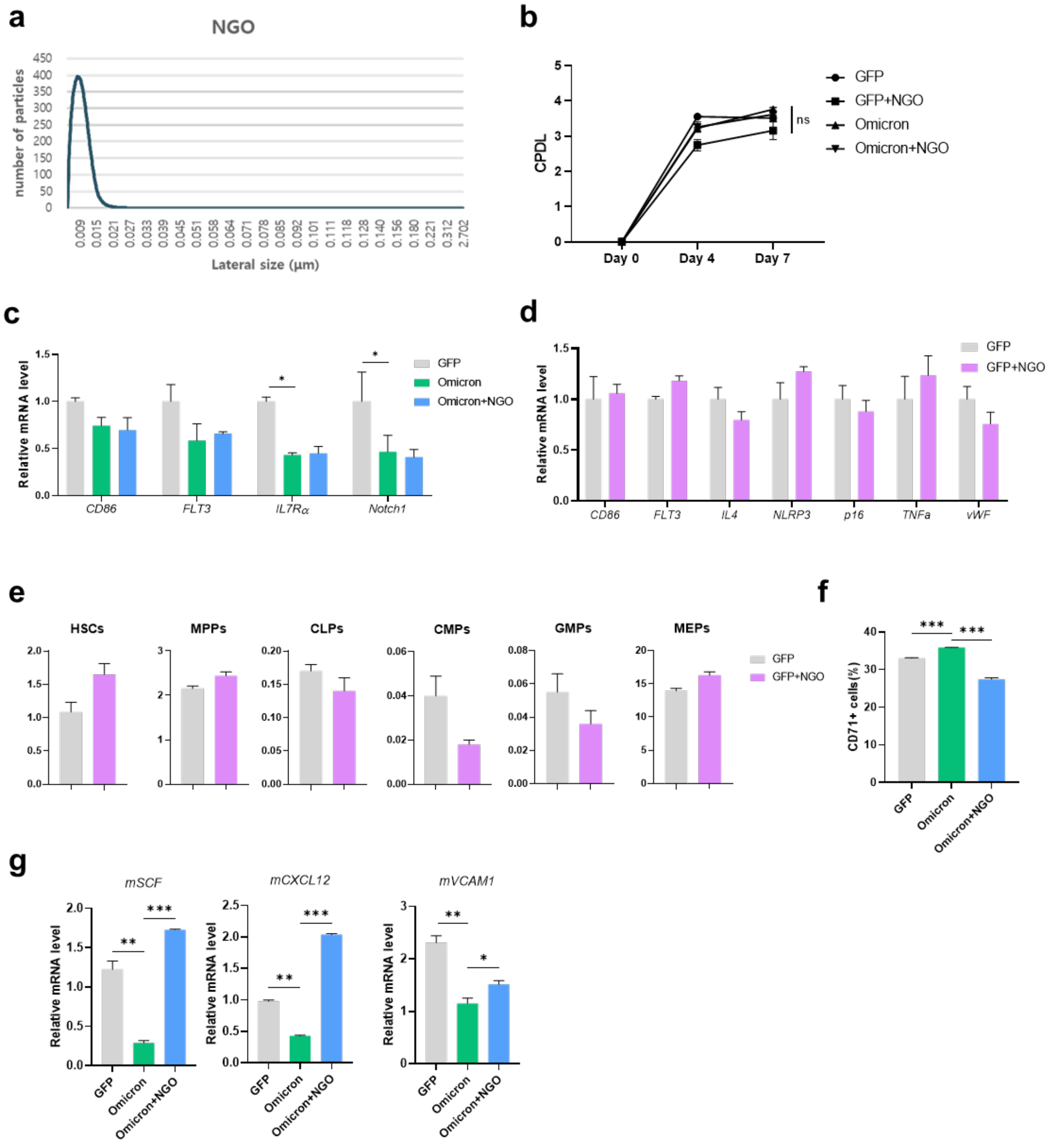

Supplementary Fig. 3. (a) Particle size distribution of NGOs. (b) CPDL of Omicron PsV-

infected HSPCs and NGO-treated HSPCs. (c) Transcriptional levels of lymphoid lineage-related genes in Omicron PsV-infected HSPCs were determined by qRT-PCR (d) Transcriptional levels of inflammaging-related genes in GFP- and NGO-treated HSPCs were determined by qRT-PCR. mRNA levels were normalized to *GAPDH*. (e) Hematopoietic lineage differentiation was investigated using lineage-specific markers and flow cytometry. Percentage HSPC lineage differentiation fractions in GFP- and NGO-treated group are shown. (f) Percentage of CD71+ RBC progenitor cells was determined by using flow cytometry (g) Transcriptional levels of BM-niche related genes in bone marrow were quantified using qRT-PCR. mRNA levels were normalized to *mGAPDH* *, $p<0.05$; **, $p<0.01$; ***, $p<0.001$. Results are shown as mean ± SEM.
